# Exposure to Perfluoroalkyl Acids and Markers of Kidney Function among Children and Adolescents Living near a Chemical Plant

**DOI:** 10.1289/ehp.1205838

**Published:** 2013-03-11

**Authors:** Deborah J. Watkins, Jyoti Josson, Beth Elston, Scott M. Bartell, Hyeong-Moo Shin, Veronica M. Vieira, David A. Savitz, Tony Fletcher, Gregory A. Wellenius

**Affiliations:** 1 Department of Epidemiology, Brown University, Providence, Rhode Island, USA; 2 Social and Environmental Health Research, London School of Hygiene and Tropical Medicine, London, UK; 3 Program in Public Health, and; 4 School of Social Ecology, University of California, Irvine, Irvine, California, USA; 5 Department of Environmental Health, Boston University School of Public Health, Boston, Massachusetts, USA

**Keywords:** adolescent, children, eGFR, kidney function, perfluoroalkyl acids, perfluorooctane sulfonate, perfluorooctanoic acid, reverse causation

## Abstract

Background: Serum levels of perfluorooctanoic acid (PFOA) have been associated with decreased renal function in cross-sectional analyses, but the direction of the association is unclear.

Objectives: We examined the association of measured and model-predicted serum PFOA concentrations with estimated glomerular filtration rate (eGFR), a marker of kidney function, in a highly exposed population (median serum PFOA, 28.3 ng/mL).

Methods: We measured serum creatinine, PFOA, perfluorooctane sulfonate (PFOS), perfluorononanoic acid (PFNA), and perfluorohexane sulfonate (PFHxS) and calculated eGFR in 9,660 children 1 to < 18 years of age at study enrollment. We predicted concurrent and historical serum PFOA concentrations using a validated environmental, exposure, and pharmacokinetic model based on individual residential histories, and used linear regression to estimate the association between eGFR and measured and predicted serum PFOA concentrations. We hypothesized that predicted serum PFOA levels would be less susceptible to reverse causation than measured levels.

Results: An interquartile range increase in measured serum PFOA concentrations [IQR ln(PFOA) = 1.63] was associated with a decrease in eGFR of 0.75 mL/min/1.73 m^2^ (95% CI: –1.41, –0.10; *p* = 0.02). Measured serum levels of PFOS, PFNA, and PFHxS were also cross-sectionally associated with decreased eGFR. In contrast, predicted serum PFOA concentrations at the time of enrollment were not associated with eGFR (–0.10; 95% CI: –0.80, 0.60; *p* = 0.78). Additionally, predicted serum PFOA levels at birth and during the first ten years of life were not related to eGFR.

Conclusions: Our findings suggest that the cross-sectional association between eGFR and serum PFOA observed in this and prior studies may be a consequence of, rather than a cause of, decreased kidney function.

Chronic kidney disease affects approximately 13% of the U.S. population, and its prevalence has been increasing over the past 20 years (Coresh et al. 2007). Chronic kidney disease is an important health problem that frequently leads to kidney failure, and is an important risk factor for cardiovascular disease (Sarnak et al. 2003; Wright and Hutchison 2009).

Perfluoroalkyl acids (PFAAs) are a class of synthetic chemicals that have been used in a wide range of commercial and industrial applications since the 1950s. Uses include stain resistant sprays for carpeting and upholstery, food contact paper coatings, nonstick cooking surfaces, and waterproofing sprays. Many PFAAs are persistent environmental pollutants (Lau et al. 2007), and > 95% of Americans participating in the 2007–2008 National Health and Nutrition Examination Survey (NHANES) had detectable concentrations of the four most common PFAAs—perfluorooctanoic acid (PFOA), perfluorooctane sulfonate (PFOS), perfluorononanoic acid (PFNA), and perfluorohexane sulfonate (PFHxS)—in their blood (Kato et al. 2011). Mean serum concentrations of PFOS and PFOA have decreased in recent years in the United States (Bartell et al. 2010; Kato et al. 2011; Olsen et al. 2012), possibly due to the cessation of production of PFOS by large U.S. producers and efforts to decrease release of PFOA into the environment. On the other hand, serum concentrations of PFNA have been increasing over the past 10–15 years (Kato et al. 2011).

Epidemiological studies suggest that exposure to PFOA and PFOS may be associated with several risk factors for kidney disease, including increased total and low-density lipoprotein (LDL) cholesterol (Costa et al. 2009; Frisbee et al. 2010; Nelson et al. 2010; Steenland et al. 2009b), increased levels of uric acid (Costa et al. 2009; Shankar et al. 2011a; Steenland et al. 2010b), increased prevalence of insulin resistance and metabolic syndrome (Lin et al. 2009), and increased risk of diabetes (Leonard et al. 2008; Lundin et al. 2009), although results have not been entirely consistent (MacNeil et al. 200; Steenland et al. 2010a). Although previous studies of populations that were occupationally or otherwise highly exposed to PFOA did not find an association between serum PFOA concentrations and blood urea nitrogen or serum creatinine (Costa et al. 2009; Emmett et al. 2006), a recent analysis of NHANES data found that serum concentrations of PFOA and PFOS were associated with decreased estimated glomerular filtration rate (eGFR) and increased odds of having an eGFR < 60 mL/min/1.73 m^2^, a clinically relevant cut point indicative of chronic kidney disease in adults (Shankar et al. 2011b).

Because NHANES is a cross-sectional survey, Shankar et al. (2011b) were unable to determine whether high levels of PFOA and PFOS in serum preceded reduced kidney function and chronic kidney disease, or vice versa. In addition, serum concentrations of PFOA and PFOS are moderately or strongly correlated in the general population (Calafat et al. 2007), making it difficult to distinguish the association with one from the other. Finally, the association between PFAA exposure and kidney function among children or adolescents has never been examined. Because chronic kidney disease in children may eventually require dialysis or kidney transplantation, and early diagnosis is a key component of successful treatment, understanding environmental risk factors is important.

Accordingly, the objective of this study was to evaluate the cross-sectional and longitudinal association between measures of PFAA exposure and kidney function among children and adolescents who were highly exposed to PFOA from contaminated water supplies, but exposed to typical U.S. levels of PFOS, PFNA, and PFHxS. We evaluated these associations within the context of the data gathered in the C8 Health Project, a large, population-based health survey of residents living in the mid-Ohio River Valley and exposed to high levels of PFOA in drinking water resulting from contamination from a local industrial facility (Frisbee et al. 2009).

## Materials and Methods

*Population and study design*. The C8 Health Project resulted from the settlement of a class-action lawsuit filed in 2002 by residents living near the DuPont Washington Works plant in West Virginia after PFOA released from the plant contaminated drinking-water supplies along the mid-Ohio River Valley, as previously described (Frisbee et al. 2009). Residents were eligible to participate in the C8 Health Project if they could document consumption of public drinking water from contaminated water districts or a small number of private wells known to be contaminated for at least 1 year between 1950 and 3 December 2004 at their primary residence, workplace, or school. Participants were enrolled in the C8 Health Project between 1 August 2005 and 31 August 2006, and all participants provided written informed consent before enrollment. An estimated 80% of then-residents of eligible water districts enrolled in the C8 Health Project. The C8 Health Project was approved by the respective institutional review boards (IRBs) associated with the principal investigators of that study (Frisbee et al. 2009). The specific analysis reported here was approved by the Brown University IRB.

On enrollment in the C8 Health Project, participants completed self-administered questionnaires on demographics, personal health history, residential history, and lifestyle habits (Brookmar Inc. 2005) and provided a venous blood sample. Information collected included sex, race (non-Hispanic white vs. other), height, weight, household income (≤ $30,000 vs. > $30,000), education (< 12 years, high school diploma or GED, some college, bachelor degree or higher), smoking status (ever vs. never) and regular exercise (yes vs. no). The complete questionnaire is available online Brookmar Inc. 2005).

*Blood sampling and laboratory methods*. Blood samples were centrifuged, divided into aliquots, and refrigerated until shipment to the laboratory for analysis. Fasting before blood sampling was not required. Serum creatinine was measured at an accredited clinical diagnostic laboratory (LabCorp, Inc., Burlington, NC) using the Jaffe reaction method. Laboratory analysis of PFAAs (Exygen Research Inc., State College, PA) has been previously described (Frisbee et al. 2009).

*Estimated historical PFOA exposure*. A unique feature of this population is that PFOA exposures were attributable predominantly to documented and well-defined pollutant releases from a specific industrial plant. Local PFOA releases to both air and surface water began in the early 1950s, increased until the late 1990s, and declined rapidly thereafter. Consequently, we used a series of linked environmental, exposure, and pharmacokinetic models in conjunction with self-reported residential histories to estimate historical PFOA exposures for all participants in the C8 Health Project, as previously described (Shin et al. 2011a). Briefly, estimated yearly PFOA releases from the plant were used along with meteorological and hydrogeological information to estimate concentrations of PFOA in air and water over time. Using residential histories linked to public water systems through a geographic information system, standard assumptions about air and water intake rates and body weight, and a PFOA half-life of 3.5 years (Olsen et al. 2007), yearly PFOA serum concentrations were estimated for each participant.

Measured serum PFOA concentrations from blood sampled at time of enrollment in the C8 Health Project (2005–2006) are not necessarily strongly correlated with historical modeled PFOA serum concentrations from several years to decades earlier because of changing concentrations in drinking water, varying water intake over time, residential mobility, and a serum half-life of about 2–4 years (Bartell et al. 2010; Olsen et al. 2007). However, the Spearman rank correlation coefficient (*r*_S_) between measured and modeled serum PFOA concentrations in 2005–2006 (at the time of the C8 Health Project survey) was 0.67 overall, and higher for participants who resided or worked in the contaminated area for at least 5 years before sample collection and provided daily public well water consumption information (*r*_S_ = 0.82) (Shin et al. 2011a, 2011b). In the current analysis, we used model-based estimates without any adjustment for measured serum PFOA concentrations (i.e., uncalibrated estimates). Because participants were not exposed to PFAAs other than PFOA via contaminated drinking water, we could not reliably model historical exposures to PFOS, PFNA, or PFHxS, and we could examine only associations between measured serum concentrations of these compounds and kidney function in cross-sectional analyses.

We restricted the current study to 9,783 study participants ≥ 1 to < 18 years of age at enrollment, with data available on serum creatinine and height to calculate eGFR, and measured serum PFOA concentrations. We calculated body mass index (BMI) *z*-score based on the 2000 Centers for Disease Control and Prevention (CDC) growth charts of BMI for age and sex (CDC 2009) using SAS (version 9.2; SAS Institute Inc., Cary, NC). We calculated eGFR using the following formula (Schwartz and Work 2009):

eGFR = *k* × height in cm/serum creatinine, [1]

where *k* is an age-specific constant equal to 0.7 for males 13–18 years of age and 0.55 otherwise. After excluding 123 subjects with implausible values of BMI *z*-score, data on 9,660 participants were available for analyses.

In a subsample of 6,060 participants who consented to research use of their residential address history, we estimated serum PFOA concentrations at the time of enrollment. Additionally, we estimated historical serum PFOA concentrations at specific time points that potentially represent critical windows of exposure, including *a*) birth, *b*) birth through 10 years of age (or current age if < 10 years old), and *c*) during the 3 years before enrollment. Cumulative measures of exposure were calculated by summing estimated yearly serum PFOA concentrations over the specified time period. Serum concentrations at birth and during the first 10 years of life could only be estimated reliably for participants born after 1989 (*n* = 4,787), because contaminant releases during this time period were the dominant source of exposure, providing the most reliable exposure estimates.

For a subsample of participants with estimated PFOA exposure at birth whose mothers also participated in the C8 Health Project (*n* = 3,527), we were able to match children to their mothers and obtain information on maternal smoking, maternal education, maternal exercise, and maternal BMI, as previously described (Mondal et al. 2012).

*Statistical analyses.* Household income was missing in 21%, BMI in 0.8%, and smoking in 0.1% of participants. We used multiple imputation to address missing covariate data. All available covariates were used to impute missing values. Twenty imputations were generated to obtain accurate information on the variability in the imputed values (Graham et al. 2007). Markov chain Monte Carlo imputation was used with 200 burn-in iterations and 100 iterations between each imputation. The parameter estimates and standard errors determined from each imputed data set were pooled into a final parameter estimate and variance using the method of Rubin (1987).

We used linear regression to evaluate the cross-sectional association between measured serum concentrations of PFOA, PFOS, PFNA, and PFHxS and eGFR among all 9,660 participants. Serum PFAA concentrations were log-transformed, modeled as linear continuous variables, and entered into separate regression models. We performed additional analyses considering quartiles of serum PFAA concentrations. Tests for linear trend were performed by assigning the median PFAA concentration in each quartile to all participants in that quartile and including the term as a continuous variable in a linear regression model. In initial models we controlled for age (natural cubic spline with 3 degrees of freedom), sex, race, smoking, and household income. In sensitivity analyses we additionally controlled for regular exercise and BMI *z*-score (natural cubic spline with 3 degrees of freedom). As a further sensitivity analysis, we additionally adjusted for total cholesterol, which could represent either a potential confounder or a causal intermediate.

We used an analogous modeling strategy to evaluate the association of predicted serum PFOA concentrations at enrollment in the C8 Health Project, at birth, from birth to 10 years of age (early life), and over the 3 years before enrollment (recent) with eGFR calculated based on serum creatinine measured at enrollment in the C8 Health Project. When considering predicted serum PFOA levels at birth we did not control for children’s smoking because none of the participants would have been smokers at birth. In sensitivity analyses, in the subset of children whose mothers also participated in the C8 Health Project, we assessed the association of predicted serum PFOA concentrations at birth with eGFR additionally controlling for maternal age (spline with 3 degrees of freedom), maternal smoking, maternal education, maternal BMI (spline with 3 degrees of freedom), and maternal regular exercise.

All analyses were conducted using SAS software (version 9.2) and R 2.12.1 (R Project for Statistical Computing, Vienna, Austria). A two-sided *p*-value of < 0.05 was considered statistically significant.

## Results

Characteristics of the 9,660 participants ≥ 1 to < 18 years of age on enrollment and with measurements available on serum PFAAs, serum creatinine, and height are shown in Table 1. These participants did not differ materially from all participants in the C8 Health Project population in this age range [see Supplemental Material, Table S1 (http://dx.doi.org/10.1289/ehp.1205838)]. Participants with measured serum PFOA in the highest quartile tended to be younger and were more likely to be male and never smokers and to have a higher household income and lower BMI *z*-score compared with other participants. Among participants included in these analyses, the median concentration of PFOA in serum was 28.3 ng/mL, with a range of 0.7–2,071 ng/mL. As expected, median concentrations of serum PFOS, PFNA, and PFHxS were lower than those of PFOA at 20.0, 1.5, and 5.2 ng/mL, respectively. Measured serum concentrations of PFOA were only weakly correlated with PFOS, PFNA, or PFHxS in serum (*r_S_* = 0.10–0.26), whereas correlations between serum concentrations of PFOS, PFNA, and PFHxS were stronger (*r*_S_ = 0.24–0.54) (see Supplemental Material, Table S2), as expected, because the predominant source of exposure to PFOA was different from that of other PFAAs. Mean eGFR (Table 1) was well within the normal ranges for children and adolescents (Hogg et al. 2003), with 0.3% of participants (*n* = 29) with an eGFR below normal for their age and sex.

**Table 1:** Characteristics of 9,660 study participants < 18 years of age at enrollment into the C8 Health Project.

Characteristic	Overall	Quartiles of serum PFOA concentrations			
		1	2	3	4
Measured serum PFOA [ng/mL (range)]	0.7–2071	0.7–< 12.8	12.8–< 28.3	28.3–< 65.4	≥ 65.4–2,071
Measured serum PFOA [ng/mL (median)]	28.3	9.1	18.6	41.3	139.2
Age [years (mean ± SD)]	12.4 ± 3.8	12.8 ± 3.7	12.6 ± 3.8	12.4 ± 3.8	12.0 ± 3.8
Female [n (%)]	4,684 (48)	1,303 (54)	1,203 (49)	1,144 (47)	1,034 (43)
White [n (%)]	9,346 (97)	2,326 (97)	2,347 (97)	2,349 (97)	2,324 (96)
Ever smoker [n (%)]	92 (1.0)	30 (1.3)	37 (1.5)	18 (0.7)	7 (0.3)
Regular exercise [n (%)]	3,939 (41)	889 (37)	922 (38)	1,068 (44)	1,060 (44)
Household income ≤ $30,000/year [n (%)]	3,679 (48)	1,249 (52)	1,217 (50)	1,089 (45)	1,007 (42)
BMI z-score (mean ± SE)	0.6 ± 1.2	0.63 ± 0.02	0.58 ± 0.03	0.56 ± 0.03	0.51 ± 0.03
eGFR [mL/min/1.73 m2 (mean ± SD)]	133.0 ± 23.9	133.0 ± 24.1	132.6 ± 22.6	133.1 ± 26	133.2 ± 22.9

*Measured serum PFAA concentrations and eGFR*. A shift from the 25th percentile to the 75th percentile of measured, natural log–transformed concentrations of PFOA in serum [interquartile range (IQR) ln(PFOA) = 1.63] was associated with a decrease in eGFR of 0.75 mL/min/1.73 m^2^ (95% CI: –1.41, –0.10; *p* = 0.02) adjusting for age, sex, race, smoking status, and household income (Table 2). Further adjustment for regular exercise and BMI *z*-score did not materially alter the results, nor did further adjustment for total cholesterol. Measured concentrations of PFOS, PFNA, or PFHxS in serum were also significantly associated with lower eGFR.

**Table 2:** Associations between serum perfluoroalkyl acid (PFAA) concentrations and estimated glomerular filtration rate (eGFR).^*a*^

PFAA	n	IQR	Model 1b		Model 2c	
			Change in eGFR (95% CI)c	p-Value	Change in eGFR (95% CI)c	p-Value
Measured						
PFOA	9,660	1.63	–0.75 (–1.41, –0.10)	0.02	–0.73 (–1.38, –0.08)	0.03
PFOS	9,660	0.64	–1.10 (–1.66, –0.53)	0.0001	–1.34 (–1.91, –0.77)	< 0.0001
PFNA	9,660	0.51	–0.83 (–1.35, –0.30)	0.002	–0.88 (–1.41, –0.36)	0.001
PFHxS	9,660	1.27	–0.95 (–1.57, –0.32)	0.003	–1.02 (–1.64, –0.40)	0.001
Estimated PFOA						
Earlyd	4,787	2.10	–0.03 (–0.99, 0.93)	0.95	–0.09 (–1.04, 0.87)	0.86
Recente	6,060	1.88	–0.04 (–0.76, 0.68)	0.91	–0.06 (–0.77, 0.65)	0.87
Enrollmentf	6,060	1.84	–0.10 (–0.80, 0.60)	0.78	–0.12 (–0.81, 0.58)	0.75
aExpressed as the mean change in eGFR per interquartile range (IQR) increase in each natural log-transformed PFAA. bModel 1: adjusted for age, sex, race, smoking, and household income. cModel 2: adjusted for model 1 covariates plus regular exercise and BMI z-score. dFirst 10 years of life, or current age if < 10 years of age. eDuring 3 years before enrollment. fAt time of enrollment (2005–2006).						

**Figure 1:**
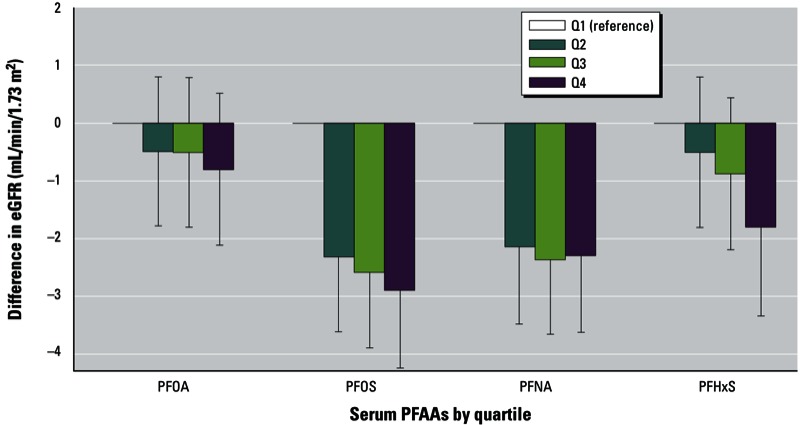
Mean change in eGFR by quartiles (Q) of measured PFAA concentrations in serum (mL/min/1.73 m^2^) adjusted for age, sex, race, smoking status, and household income. Error bars represent 95% CIs; *p*-values for trend = 0.30 for PFOA, 0.0001 for PFOS, 0.005 for PFNA, and 0.004 for PFHxS.

Considering quartiles of measured serum PFAA concentrations, eGFR decreased monotonically with increasing PFOA, PFOS, and PFHxS levels, adjusting for age, sex, race, smoking status, and household income (Figure 1). This association was strongest with serum concentrations of PFOS, with a decrease in eGFR of 2.3, 2.6, and 2.9 mL/min/1.73 m^2^ for the second, third, and fourth quartile of serum PFOS, respectively, compared with the lowest quartile. There was a statistically significant linear trend between eGFR and PFOS (*p*_trend_ across quartiles = 0.0001), PFNA (*p*_trend_ = 0.005), and PFHxS (*p*_trend_ = 0.004), but not PFOA (*p*_trend_ = 0.30).

*Estimated serum PFOA concentrations and eGFR*. Predicted serum PFOA concentrations at the time of enrollment were not associated with eGFR, nor were predicted historical serum PFOA concentrations during the first 10 years or life, and in the 3 years before enrollment (Table 2). Additionally, predicted historical serum PFOA concentrations at the time of birth were not associated with eGFR (Table 3). Additional control for maternal factors did not materially alter the association between predicted serum PFOA concentrations at birth and eGFR. Model-predicted serum PFOA concentrations at the time of enrollment, in the 3 years before enrollment (recent exposure), during the first 10 years of life (early exposure), and at birth were moderately to strongly correlated with one another (Table 4).

**Table 3:** Associations between estimated serum PFOA concentrations at birth and eGFR.^*a*^

Covariates	IQR	n	Change in eGFR (95% CI)	p-Value
PFOA at birth				
Model 1b	1.83	4,787	–0.11 (–0.96, 0.74)	0.80
Model 2c	1.83	3,527	–0.05 (–1.02, 0.91)	0.91
Model 3d	1.83	3,527	–0.01 (–0.96, 0.98)	0.99
aExpressed as the mean change in eGFR per interquartile range (IQR) increase in estimated, natural log-transformed, serum PFOA concentrations at birth. bModel 1: adjusted for age, sex, race, and household income. cModel 2: adjusted for model 1 covariates plus maternal age, maternal smoking, and maternal education. dModel 3: adjusted for model 2 covariates plus maternal exercise and maternal BMI.				

**Table 4:** Spearman correlation coefficients among measured and historical estimates of serum PFOA concentrations.

Serum PFOA	Measured at enrollment	Predicted at enrollment	Predicted at birth	Predicted early life	Predicted recent
Measured at enrollment	1.00
Predicted at enrollment	0.73a	1.00
Predicted at birth	0.43b	0.53b	1.00
Predicted early life	0.66b	0.87b	0.68b	1.00	
Predicted recent	0.73a	0.997a	0.53b	0.88b	1.00
aAmong 6,060 subjects. bAmong 4,787 subjects born in 1990 or later.					

## Discussion

In this large, community-based study, participants had a wide range of exposures to PFOA via contaminated drinking water, but background exposure to the three other common PFAAs that we measured, resulting in weak correlations of PFOA with PFOS, PFNA, and PFHxS (*r*_S_ = 0.10–0.26). This allowed us to evaluate the association between serum PFOA and kidney function with minimal concerns of potential confounding by co-exposure to other PFAAs. We also had the unique opportunity to evaluate associations between estimates of historical serum PFOA levels and kidney function using a detailed exposure model previously developed in this population (Shin et al. 2011a). We found that measured serum PFOA levels were associated with decreased eGFR, but this association was not found when we used predicted serum PFOA concentrations as a measure of exposure.

The results of our cross-sectional analyses based on measured serum PFOA levels are generally consistent with a study by Shankar et al. (2011b) of adult NHANES participants from 1999–2008, which reported cross-sectional associations between serum PFOA and PFOS levels and decreased eGFR, as well as increased odds of chronic kidney disease (defined as eGFR < 60 mL/min/1.73 m^2^). Although NHANES participants had much lower median serum PFOA levels (4.1 ng/mL) compared with our highly exposed population (28.3 ng/mL), the magnitude of the association estimated for the NHANES population was larger (–5.7 mL/min/1.73 m^2^; 95% CI: –7.9, –3.5 between the first and fourth quartile of serum PFOA compared with –0.80 mL/min/1.73 m^2^; 95% CI: –2.12, 0.52 observed in the present analysis). This may be explained, at least in part, by the difference in the magnitude of exposure between the two study populations. For example, those in the 75th percentile of serum PFOA in the NHANES population would be included in the lowest quartile of our population. Although the dose–response relationship between serum PFOA and eGFR appeared to be approximately linear at the lower serum PFOA concentrations reported among NHANES participants (Shankar et al. 2011b), our results suggest that, if in fact increased serum PFOA levels cause decreased kidney function, the dose–response curve may flatten at higher PFOA concentrations (e.g., saturation). The difference in ages between the two populations could also explain differences in the reported associations. GFR changes throughout childhood and adolescence, the developmental periods studied here (1 to < 18 years), whereas Shankar et al. (2011b) studied adults ≥ 20 years of age.

**Figure 2:**
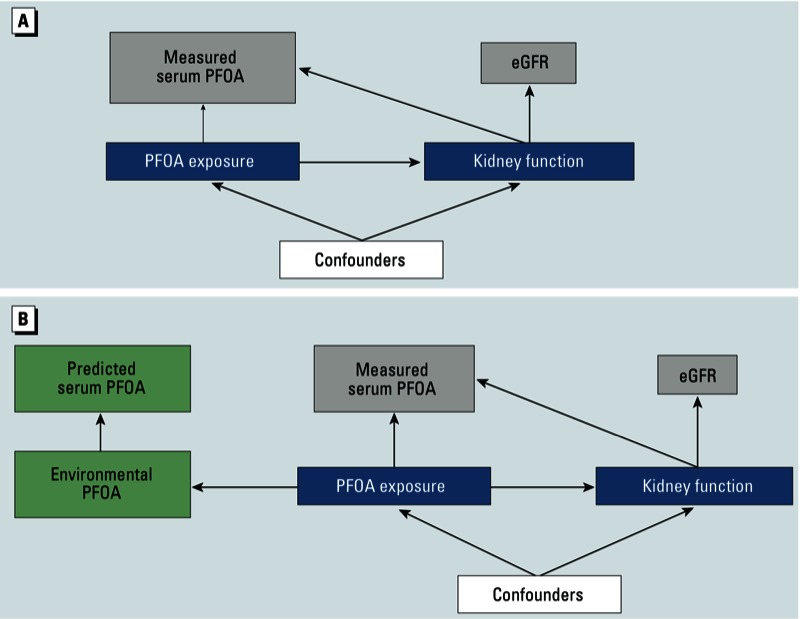
Causal diagram illustrating potential for direct and reverse causation. (*A*) The causal effect of the biologically effective dose of PFOA (PFOA exposure) on kidney function was approximated by relating serum levels of PFOA (an imperfect marker of the biologic effective dose) to eGFR (an imperfect marker of kidney function), controlling for common causes of PFOA exposure and kidney function, which may confound the association. Reverse causation occurs when kidney function alters our marker of exposure, represented by the arrow from kidney function to serum PFOA. (*B*) Biological exposure is determined by environmental levels of PFOA, which can also be used to predict serum levels of PFOA. Because kidney function does not affect predicted PFOA, analyses using predicted PFOA cannot represent reverse causation.

Even in the absence of confounding or other sources of bias, it would not be possible to determine whether serum PFAA levels cause, or are a result of, decreased kidney function, or some combination of the two, based on a cross-sectional study. The causal directed acyclic graph shown in Figure 2 illustrates the potential for reverse causation (Hernan and Cole 2009). We are interested in estimating the causal effect of the biologically effective dose of PFOA on kidney function, which we approximate by relating serum levels of PFOA (an imperfect marker of the biologically effective dose) to eGFR (an imperfect marker of kidney function), controlling for common causes of PFOA exposure and kidney disease that may confound the association. Reverse causation occurs when kidney function (the outcome) alters our chosen marker of exposure (serum PFOA).

Decreased GFR could plausibly lead to increased serum concentrations of PFAAs. Pharmacokinetic studies in animals have demonstrated that PFOA and PFOS are distributed primarily in serum, kidney, and liver (Lau et al. 2007) and are excreted by the kidneys without undergoing biotransformation (Han et al. 2012). In humans, PFOA is secreted from the blood into urine through the apical membrane of the proximal tubule cells and reabsorbed back into the blood through the basolateral membrane via uptake and efflux transport proteins (Han et al. 2012). However, because a small fraction of PFAAs are not reabsorbed in the kidney (Han et al. 2012), a decreased rate of filtration through the glomerulus (i.e., decreased GFR) could potentially result in slower elimination and a consequent increase in serum PFAAs over time. In addition, decreased GFR could also decrease the rate of travel through the renal tubules, potentially increasing the time available for PFAA reabsorption and potentially increasing concentrations of PFAAs in serum. Finally, people with decreased kidney function may sometimes experience increased thirst (National Kidney Foundation 2008), which in this population, if associated with increased intake of contaminated water, could also lead to increased PFOA in serum.

On the other hand, animal studies suggest that the kidney could be a target organ for effects of PFAA exposure. Toxicological studies have found changes in kidney histology and organ weights among rodents exposed to PFOA (Cui et al. 2009; Kennedy et al. 2004). *In vitro* studies using several cell types have found that PFAA exposure increases the permeability and fluidity of cell membranes (Hu et al. 2003; Qian et al. 2010), which are similar to changes seen in acute kidney injury in rats (Sutton 2009).

To evaluate the question of whether PFOA in serum causes decreased kidney function or vice versa, we evaluated the association between predicted serum PFOA concentrations and eGFR measured upon enrollment. As illustrated in Figure 2B, biologic exposure is determined by environmental levels of PFOA, which can also be used to predict serum levels of PFOA. Because reduced kidney function cannot affect predicted serum PFOA concentrations, analyses using the predicted levels as the exposure measure are not susceptible to the problem of reverse causation. Specifically, because subject-specific measures of water intake or renal clearance were not used in our prediction models, these factors cannot influence predicted serum PFOA concentrations.

We observed an association between measured, but not predicted, serum PFOA. One possible explanation for these results is that reduced kidney function leads to higher measured serum levels of PFOA. Under this scenario, our results could be equivalently interpreted as showing that a 1-unit increase in eGFR is associated with a 2.17-unit decrease in measured ln(PFOA) in serum among the population studied here, adjusting for all the same potential confounders.

Our finding that PFOA was less strongly associated with kidney function than other PFAAs [Figure 1; see also Supplemental Material, Table S3 (http://dx.doi.org/10.1289/ehp.1205838)] supports this reverse causality hypothesis. To see this, recall that in this population there was large variability in individual PFOA exposures (driven primarily by contaminant levels in the local water supply) but much less variation in individual exposures to other PFAAs. In this setting, and assuming that kidney function does indeed influence serum levels of all PFAAs, then one would expect small variations in kidney function to have less of an impact on measured serum PFOA levels than on measured serum levels of other PFAAs. Thus, if kidney function does influence serum levels of PFAAs, in this population we would expect to find that PFOA would be less strongly associated with kidney function than other PFAAs—exactly the pattern of results observed in this study.

Similarly, reverse causation could potentially also explain the difference in the magnitude of association between kidney function and PFOA reported by Shankar et al. (2011b) and the cross-sectional association reported here. For example, if the association observed by Shankar et al. (2011b) in the general population was attributable solely to metabolic differences (reverse causation), one would expect that association to be attenuated in our population, where environmental exposure to PFOA varied widely across individuals residing in different water districts.

An alternative explanation for our observation that kidney function was associated with measured, but not predicted, serum PFOA is that our predictions of historical PFOA serum levels might be highly misclassified. Although we cannot directly address this possibility, we note that predicted PFOA serum concentrations at the time of enrollment were strongly correlated with measured levels of serum PFOA (*r*_S_ = 0.73). Additionally, predicted historical levels of serum PFOA have been associated with other health outcomes in this population (Lopez-Espinosa et al. 2011; Savitz et al. 2012).

Finally, it is possible that the association between serum PFOA and kidney function reflects causal effects in both directions (i.e., decreased kidney function causes increased PFOA in serum and increased serum PFOA causes decreased kidney function), potentially depending on the magnitude of exposure or characteristics of the exposed population.

Our study has additional limitations. First, information on residential history and potential confounders was self-reported, inevitably leading to some misclassification and potentially resulting in residual confounding. However, in contrast to the general population, exposure to PFOA in this population was driven mostly by residential water district, and was only weakly correlated with markers of socioeconomic status and personal behaviors (Steenland et al. 2009a). Second, although our exposure model allowed us to predict serum PFOA levels at birth or during early life, no historical information was available about potential confounders. However, most known potential confounders in this population are either time-invariant (e.g., sex, race) or likely to vary only slowly over time (e.g., household income). Third, because the number of participants with an eGFR suggestive of kidney disease was small, we were unable to make comparisons between those with and without kidney disease. Last, given the unique nature of exposures in this study population, our results may not be generalizable to other populations, those with lower exposures, or older individuals. On the other hand, our study has a number of strengths, including a large population with relatively well characterized exposures to high levels of PFOA, background levels of other PFAAs, and the availability of a prediction model of historical exposures that is unlikely to be influenced by kidney function.

## Conclusions

Although we observed an association between measured serum PFOA levels and a marker of reduced kidney function in children and adolescents, our findings highlight the possibility that this association and resulting increased concentrations of PFOA may, at least in part, be a consequence, rather than a cause, of decreased kidney function. We were not able to predict historical serum concentrations of other common PFAAs as we could for PFOA, but we hypothesize that the observed cross-sectional associations of measured serum concentrations of PFOS, PFNA, and PFHxS with decreased kidney function may also be attributable at least partly to reverse causation. Further research in the context of prospective cohort studies is needed to clearly establish whether the observed associations of eGFR with PFAAs may be the cause or result of reduced renal function, or a combination of both.

## Supplemental Material

(418 KB) PDF
