# Supplementary material for: Exposure to Perfluoroalkyl Acids and Markers of Kidney Function among Children and Adolescents Living near a Chemical Plant
Source: Environ Health Perspect. 2013 Mar 11;121(5):625–30. doi: 10.1289/ehp.1205838 (PMC3673193; doi:10.1289/ehp.1205838)
Supplement: (418 KB) PDF [file ehp.1205838.s001.pdf]

Supplemental Material

Exposure to Perfluoroalkyl Acids and Markers of Kidney Function among Children and Adolescents Living Near a Chemical Plant

Deborah J. Watkins, Jyoti Josson, Beth Elston, Scott M. Bartell, Hyeong-Moo Shin, Veronica M. Vieira, David A. Savitz, Tony Fletcher, Gregory A. Wellenius

Table of Contents **Page**

Table S1: Population characteristics 2

Table S2: Spearman correlations between serum concentrations of different PFAAs measured at enrollment (2005-2006) 3

Table S3: Mean change in eGFR by quartiles of measured PFAA concentrations 3

Table S1: Population characteristics

Characteristic	All C8 Health Project Participants aged 1 to <18 years (n = 12,476)	C8 Health Project Participants included in current analysis (n = 9,660)	C8 Health Project Participants included in current analysis with predicted historical serum PFOA concentrations (n= 6,060)
Age, years, mean ± SD	11.1 ± 4.5	12.4 ± 3.8	12.6 ± 3.8
Female, N (%)	6113 (49)	4684 (48)	2968 (49)
White, N (%)	12059 (97)	9346 (97)	5841 (96)
Ever Smoker, N (%)[a]	94 (0.8)	92 (1.0)	77 (1.3)
Regular Exercise, N (%)	4576 (37)	3939 (41)	2500 (41)
Household Income ≤$30,000/year, N (%)[b]	4774 (48)	3679 (48)	2298 (48)
BMI z-score, mean ± SE[c]	0.6 ± 1.5	0.6 ± 1.2	0.6 ± 1.2
eGFR, ml/min/1.73m^2, mean ± SD[d]	132.9± 24.0	133.0± 23.9	132.3 ± 23.0

Participants in total population missing:[a] smoking status = 11 (0.01%); [b] household income = 2,622 (21.0%); [c] BMI = 1,312 (10.5%);

[d] eGFR = 2,700 (21.6%).

Table S2: Spearman correlations between serum concentrations of different PFAAs measured at enrollment (2005-2006)

PFAA	PFOA	PFOS	PFNA	PFHS
PFOA	1			
PFOS	0.26	1		
PFNA	0.10	0.45	1	
PFHS	0.25	0.54	0.24	1

Table S3. Mean change in eGFR by quartiles of measured PFAA concentrations in serum (mL/min/1.73^2) adjusted for age, sex, race, smoking status, and household income.

	PFOA	95% CI	PFOS	95% CI	PFNA	95% CI	PFHxS	95% CI
Quartile 1	ref		ref		ref		ref	
Quartile 2	-0.49	(-1.78, 0.80)	-2.32	(-3.62, -1.03)	-2.14	(-3.48, -0.81)	-0.50	(-1.81, 0.80)
Quartile 3	-0.51	(-1.80, 0.79)	-2.58	(-3.89, -1.28)	-2.37	(-3.66, -1.08)	-0.87	(-2.19, 0.44)
Quartile 4	-0.80	(-2.12, 0.52)	-2.89	(-4.24, -1.54)	-2.29	(-3.62, -0.96)	-1.80	(-3.34, -0.25)
p-value for trend		0.30		0.0001		0.005		0.004
